# Management of lung nodules newly found by virtual-assisted lung mapping: a case report

**DOI:** 10.1186/s40792-017-0327-x

**Published:** 2017-03-28

**Authors:** Masahiro Yanagiya, Masaaki Sato, Hideki Kuwano, Kazuhiro Nagayama, Jun Nakajima

**Affiliations:** Department of Thoracic Surgery, University of Tokyo, Graduate School of Medicine, 7-3-1 Hongo, Bunkyo-ku, Tokyo, 113-8655 Japan

**Keywords:** Thoracic surgery, Metastatic pulmonary tumor, Marking, Thoracoscopy, Bronchoscopy

## Abstract

**Background:**

Virtual-assisted lung mapping is a novel bronchoscopic lung marking technique that uses virtual images to perform multiple concurrent dye marking of barely palpable pulmonary tumors. Subsequent chest computed tomography is required to confirm the locations marked. We here report a patient in whom computed tomography after virtual-assisted lung mapping unexpectedly revealed additional tiny pulmonary nodules.

**Case presentation:**

A 64-year-old woman with a history of renal cell carcinoma presented with two pulmonary nodules suspicious of metastases from renal cell carcinoma. Because we anticipated that the nodules would be difficult to palpate intraoperatively, we performed virtual-assisted lung mapping prior to attempting to resect them. Computed tomography after mapping unexpectedly detected two additional nodules. Although the existing markings did not relate to the newly found nodules, we used imaginary auxiliary lines and anatomical landmarks to extend the lung map to incorporate the unexpected nodules. The additional nodules were successfully resected by thoracoscopic wedge resection. Pathologic examination identified all nodules as metastases from renal cell carcinoma, and the surgical margins were negative.

**Conclusions:**

Imaginary auxiliary lines and anatomical landmarks extended the existing lung map of virtual-assisted lung mapping, enabling resection of unexpected pulmonary nodules found in post-mapping computed tomography images.

## Background

Small pulmonary nodules can be found in computed tomography (CT) images taken just before surgery, posing a challenge for surgeons. Virtual-assisted lung mapping (VAL-MAP), a novel lung marking technique for facilitating resection of barely palpable pulmonary tumors [1], is a preoperative bronchoscopic procedure in which virtual images are used to make multiple concurrent markings by dye injection on the surface of a lung. A recent multi-center prospective study that accumulated 500 cases of VAL-MAP demonstrated safety, a high rate of successful resection (nearly 99%), and excellent reproducibility among different centers [2], suggesting that this procedure could become a standard approach to barely identifiable tumors in the near future.

Part of the procedure required for successful use of VAL-MAP is performing chest computed tomography (CT) 2–3 h after VAL-MAP to confirm the locations of the markings and construct three-dimensional images of the markings and tumors to plan surgery [3]. However, we recently encountered a patient whose post-VAL-MAP CT showed unexpected additional tiny pulmonary nodules, posing a challenge for the surgeons. This report describes the successful use of imaginary auxiliary lines and anatomical landmarks in combination with VAL-MAP to achieve complete resection of all nodules, including the unexpectedly identified additional ones.

## Case presentation

**Fig. 1:**
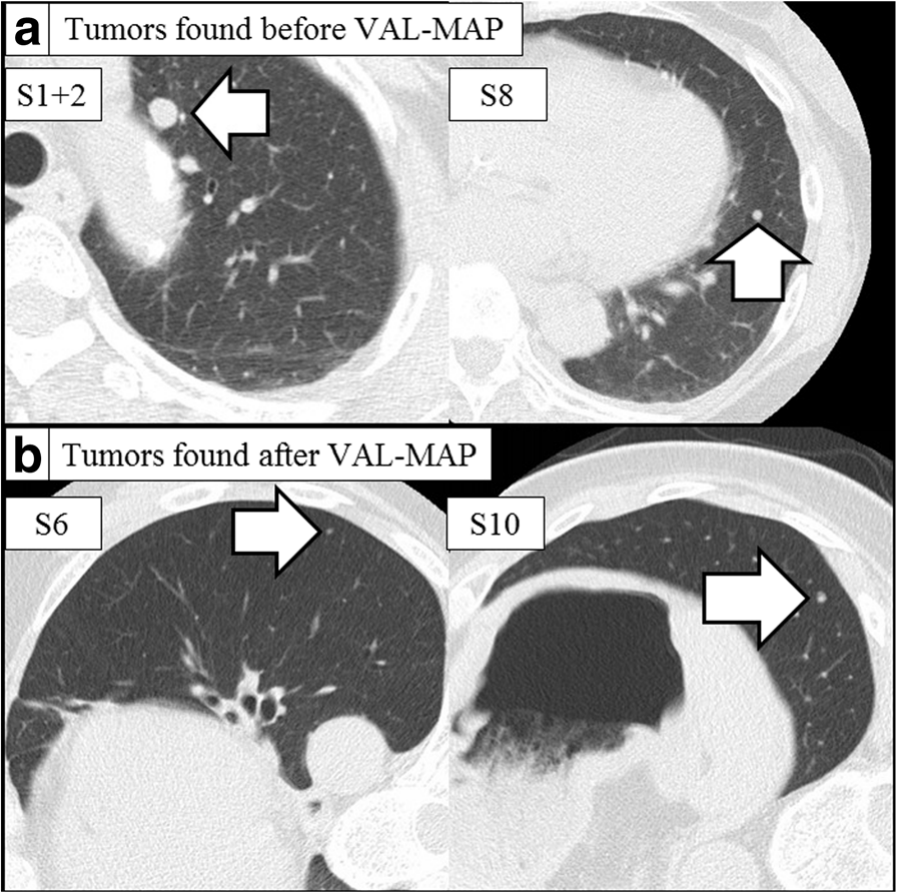
Chest computed tomography images of pulmonary nodules. **a** Chest computed tomography (CT) images taken before bronchoscopic marking revealing an 8-mm nodule located in S1 + 2 and a 5-mm nodule in S8. **b** Images taken just after bronchoscopic marking showed two unexpected tumors; a 2-mm nodule located in S6 and a 3-mm nodule in S10. The chest computed tomography was taken in a right decubitus position, facilitating detection of tiny nodules that may have been hidden by the effect of gravity in the pre-VAL-MAP CT scan, which was performed with the patient lying supine

A 64-year-old woman was diagnosed as having pulmonary metastases from renal cell carcinoma. She had undergone surgical resections of bilateral renal cell carcinomas 4 years and 1 year previously. A chest CT revealed two nodules in the left lung suspicious of metastases (Fig. 1a) with no evidence of other malignancies. Thoracoscopic resections were planned using VAL-MAP. In accordance with the standard protocol, another CT was taken after VAL-MAP with the patient in a right decubitus position [4] to maximize inflation of the posterior part of the lung and thus assist visualization of the dorsally placed markings.

**Fig. 2:**
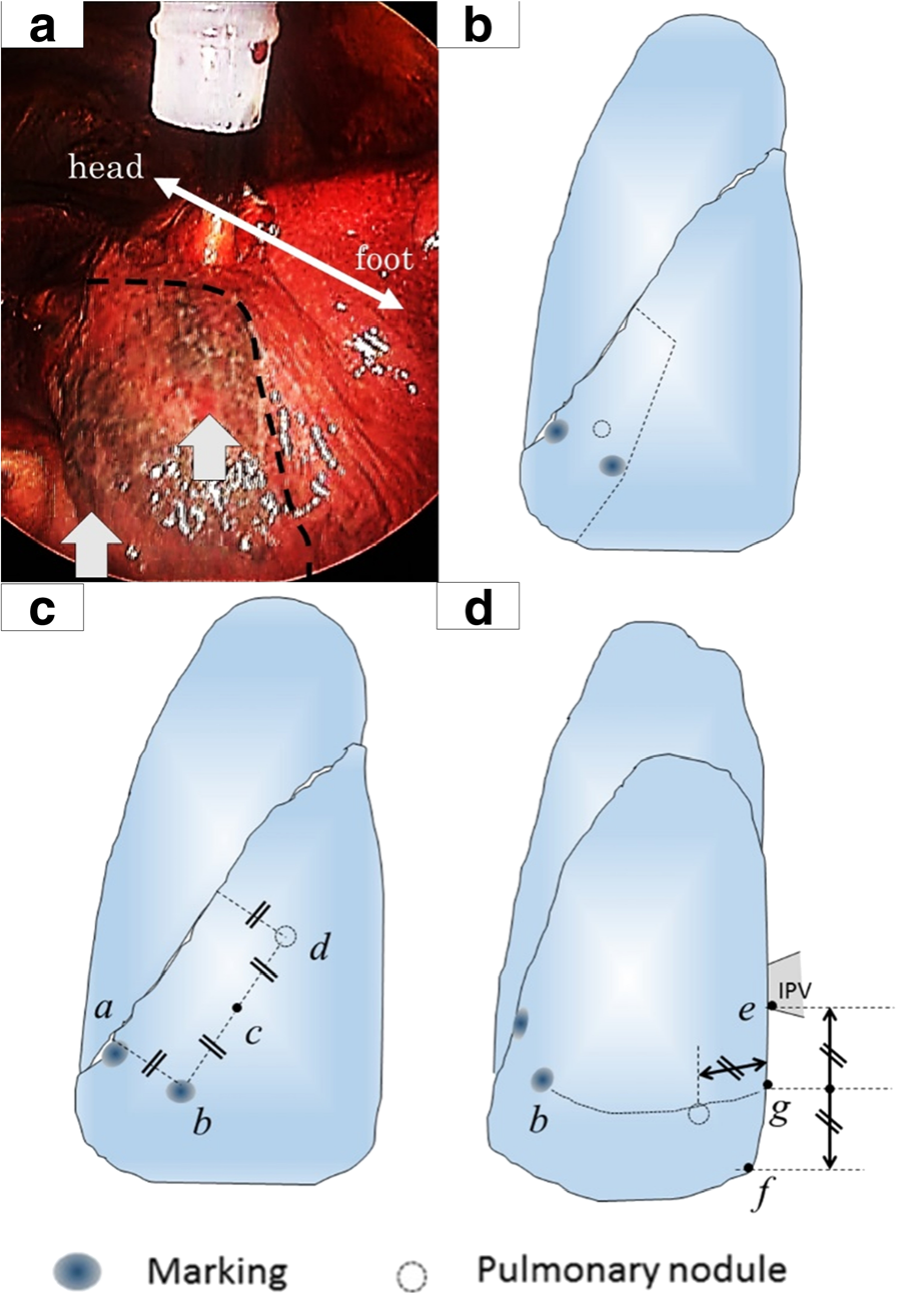
Intraoperative view of markings and three-dimensional images. **a** An intraoperative thoracoscopic view of the lung showing two markings (*white arrowheads*) indicating the location of the tumor in S8 and resection lines for S8 segmentectomy (*interrupted line*). **b** A three dimensional view showing the S8 tumor, two markings and resection lines for S8 segmentectomy. **c** A three-dimensional view showing the newly found S6 nodule, two markings, and imaginary auxiliary lines. **d** A three-dimensional view showing the newly found S10 nodule, two markings, and the inferior pulmonary vein

However, this CT unexpectedly revealed two additional suspicious tiny nodules in the left lower lobe that had presumably been hidden by the effect of gravity in pre-VAL-MAP CT, which was taken 3 weeks before the VAL-MAP procedure (Fig. 1b). A three-dimensional post-VAL-MAP CT image was constructed to display the markings and all nodules. Markings were placed near the lesion in S8 (Fig. 2), which was distant from the additional nodules.

To address this challenge, imaginary auxiliary lines and anatomical landmarks were constructed to extend the lung map to include the unexpected nodules located in S6 and S10 (Fig. 2). Particular attention was paid to the ratio of certain measurements, which would not change greatly after deflation of the lung. As shown in Fig. 2, imaginary auxiliary lines were drawn in complete deflation state, based on the markings and anatomical landmarks such as the pulmonary ligament and inferior pulmonary vein. To enable location of the newly found nodules in S6, an auxiliary line was drawn vertically from the interlobar fissure to the inferior marking (point a to point b in Fig. 2c). From point b, another auxiliary line was drawn in parallel with the interlobar fissure (point b to point d in Fig. 2c), its length being double that between points a and b (i.e., point c is the midpoint between points b and d; the nodule in S6 was located close to point d in Fig. 2c).

To enable location of the additional nodules in S10, an auxiliary line was drawn in complete deflation state, horizontally from point g in Fig. 2d between the inferior border of the inferior pulmonary vein (point e in Fig. 2d) and the inferior edge of the pulmonary ligament (point f in Fig. 2d); the nodule in S10 was located on this line approximately the same distance away from point g in Fig. 2d as the distance between points e and g in Fig. 2d. The bronchoscopic marking (point b in Fig. 2d) was also located on the same line and used as confirmation of the location of the nodule.

Even though additional lesions were barely palpable, they were successfully located and thoracoscopically resected by wedge resections with the aid of the imaginary auxiliary lines and anatomical landmarks (Fig. 2c, d) in addition to the scheduled procedure. There were no postoperative complications. Pathologic examination revealed that all nodules were metastases from renal cell carcinoma and all surgical margins were negative. The patient remains alive without recurrence 12 months after surgery.

### Discussion

We successfully resected pulmonary nodules discovered unexpectedly just after VAL-MAP. Such newly found nodules may well be encountered in post-VAL-MAP CT images, some because they became large enough to detect during the interval between pre- and post-VAL-MAP CTs, particularly in the case of metastatic lung tumors, and others, more likely, being tiny nodules that remained undetected in the pre-VAL-MAP CT because of positional differences. To better visualize dorsally placed markings, post-VAL-MAP CTs are routinely performed with the patient in a decubitus position [4]. In our slightly obese patient, the additional nodules appear to have been hidden by the effect of gravity in the pre-VAL-MAP CT, which was performed with the patient lying supine. Although the incidence of such cases is unknown, this report highlights the issue of patients’ positions during chest CT screening for prior malignancy and during VAL-MAP.

Management of such unexpected tiny pulmonary nodules poses the following two problems: whether surgical intervention is still appropriate and, if so, how best to locate the newly found nodules intraoperatively. Surgical resection of pulmonary metastasis is indicated provided the primary lesion is completely controlled, metastases have only been detected in the lungs, and the patient can tolerate surgery [5]. We decided to resect all tumors because our patient satisfied all these criteria.

Then next question was whether to take the patient back to the bronchoscopy suite to add more dye markings for the newly found lesions. However, we considered we would be able to resect the nodules if we extended the existing lung map by adding anatomical landmarks and imaginary auxiliary lines on three-dimensional images rather than adding dye markings, as reported previously [6]. Anatomical landmarks such as the pulmonary ligament are promising guides to detecting barely palpable pulmonary nodules. Moreover, the imaginary auxiliary lines constructed by connecting dye markings and anatomical landmarks are also important guides to detecting such nodules [6]. The present report demonstrates that imaginary auxiliary lines do indeed assist intraoperative location of unexpected tumors. Because the additional tumors were <5 mm in diameter and both were >5 mm from the pleural surface, the probability of successfully locating them without markings was extremely low [7]. The auxiliary lines combined with VAL-MAP and anatomical landmarks enabled complete resection of all nodules with sufficient surgical margins, as is important in oncological surgery [8]. If we had failed to palpate the additional tumors with the thoracoscopic approach, we would have converted to mini-thoracotomy for better palpation with two fingers rather than one through a thoracic port. If even this strategy had failed, we would have planed the secondary operation after growing them up.

Importantly, effective use of auxiliary lines requires consideration of the impact of intraoperative deflation of the lung. Because the distances between lesions and landmarks change when the lung is collapsed, ratios calculated from points on multiple imaginary auxiliary lines are useful. In this case, for example, the distance from a marking to the interlobar fissure was almost the same as that from the nodule in S6 to that fissure (Fig. 2b). Indeed, we have drawn the auxiliary lines in complete deflation state. The proportion between these distances would remain similar in the deflated lung.

## Conclusions

In summary, we successfully achieved complete resection of unexpected pulmonary nodules found after VAL-MAP marking with the aid of imaginary auxiliary lines and anatomical landmarks.

## Abbreviations

CT: Computed tomography
VAL-MAP: Virtual-assisted lung mapping
